# TRPV1 and TRPM8 in Treatment of Chronic Cough

**DOI:** 10.3390/ph9030045

**Published:** 2016-07-28

**Authors:** Eva Millqvist

**Affiliations:** Department of Allergology, Institution of Internal Medicine, The Sahlgrenska Academy at University of Gothenburg, 413 45 Gothenburg, Sweden; eva.millqvist@medfak.gu.se; Tel.: +46-708-43-3819; Fax: +46-500-43-3819

**Keywords:** chronic cough, TRPV1, TRPM8, TRP antagonists, desensitization

## Abstract

Chronic cough is common in the population, and among some there is no evident medical explanation for the symptoms. Such a refractory or idiopathic cough is now often regarded as a neuropathic disease due to dysfunctional airway ion channels, though the knowledge in this field is still limited. Persistent coughing and a cough reflex easily triggered by irritating stimuli, often in combination with perceived dyspnea, are characteristics of this disease. The patients have impaired quality of life and often reduced work capacity, followed by social and economic consequences. Despite the large number of individuals suffering from such a persisting cough, there is an unmet clinical need for effective cough medicines. The cough treatment available today often has little or no effect. Adverse effects mostly follow centrally acting cough drugs comprised of morphine and codeine, which demands the physician’s awareness. The possibilities of modulating airway transient receptor potential (TRP) ion channels may indicate new ways to treat the persistent cough “without a reason”. The TRP ion channel vanilloid 1 (TRPV1) and the TRP melastin 8 (TRPM8) appear as two candidates in the search for cough therapy, both as single targets and in reciprocal interaction.

## 1. Introduction

### 1.1. Chronic Cough

Coughing by humans is a necessary protective mechanism to prohibit food and foreign substances from reaching and harming the lower airways. However, coughing is also a symptom that signals attention in the diagnosis of several diseases.

Coughing is one of the most common symptoms for which patients consult a doctor in the western world, and the most usual cause is a common cold with associated cough [1,2,3]. However, when coughing is not effective enough to “clear” the airways from phlegm and mucus, it can lead to a variety of pathological conditions like atelectasis, bronchiectasis, pneumonia, lung abscesses, and pulmonary scarring [4].

The definition of coughing varies in literature, but daily coughing, when it lasts for more than two months, is, in general, regarded as chronic [5]. In addition, epidemiologic information on the prevalence of chronic cough varies, and it is reported that up to 20% of the adult population suffers from long lasting cough [2,6] with the condition related to a negative influence on quality of life and social activities [7,8,9].

When clinical tests do not give any indication of well-known causes for coughing like airway infection, asthma, post-nasal drip, chronic obstructive pulmonary disease (COPD), gastroesophageal reflux disease, cancer, alveolitis, heart failure or medication with angiotensin-converting enzyme (ACE) inhibitors, there is still a group of patients left over with chronic cough without a specific diagnosis having an ongoing cough, often refractory to available cough medications. In the present review, such patients will be referred to as having chronic idiopathic cough (CIC). How common this condition is has, however, been debated [5,10]. A similar group of patients with airway symptoms induced by environmental irritants, reporting problems with chronic coughing, chest discomfort, dyspnea, rhinitis, and eye irritation, has been identified [11,12]. The symptoms mimic asthma, but asthma-specific tests are negative. These patients have an increased cough reaction to inhaled capsaicin (the active compound of chili peppers), a tasteless and odorless substance that stimulates sensory nerves, and the provoked cough reflects sensory nerve reactivity [13]. Such airway symptoms are interpreted as airway sensory hyperreactivity (SHR). Cigarette smoke, car exhaust, perfumed products, and cold air are some of the triggers for SHR symptoms [11]. SHR affects more than 6% of the adult population in Sweden, mainly women, according to a population-based epidemiologic study [12]. In most cases, the patients could also be diagnosed with CIC [14] or the recently established cough hypersensitivity syndrome (CHS) [15]. This syndrome includes several airway conditions characterized by easily evoked cough reflex and increased cough sensitivity to inhaled capsaicin [15,16,17,18]. There was a high degree of agreement in a recent article reporting how opinion leaders in cough research regarded the suggestion of CHS as a cause underlying the cough etiology in CIC [19], and it is today, together with some forms of itch and pain [20], regarded as a possible neuropathic disease following neural injuries from various inflammatory, infective, and irritative influences [21,22,23].

### 1.2. The Medical Problem of Treating CIC

Patients with pronounced CIC have, with little success in most cases, frequently tried a variety of asthma, COPD, and cough medications. The international market for over the counter cough medication is huge, reaching several billion euros [24], though there are few scientific data supporting the effects of these products [25]. Whereas centrally acting medications like codeine and morphine can decrease coughing temporarily, they are connected with well-known adverse effects like drowsiness, difficulty concentrating, symptoms of the gall bladder, and constipation. In addition, there is a risk of habituation or abuse. Recent research indicates that pregabalin and gabapentin may have a role in treating severe CIC [23,26], though it is necessary to be aware of potential adverse reactions. There is an unmet clinical need for new, safe, and effective cough therapies with few adverse effects [10].

## 2. TRP Ion Channels in the Airways

The TRP ion channels can be found abundantly in the airways, as in most of the human organ systems and have during the last decades been important for studying multiple organ systems and their interaction with the environment [13,27]. Many of these ion channels are present in primary airway sensory neurons, some of which transmit nociceptive information to the brain. Furthermore, TRPV1 channels are expressed not only in sensory neurons but also in airway smooth muscle and epithelial cells [28,29], and some evidence suggests that TRPV1 has functional roles in the immune system [30,31].

The TRPV1 ion channel together with the later identified transient ion channel ankyrin 1 (TRPA1) have important functions in airway chemo-sensation and reflex control regarding temperature, osmolarity and oxidant stress [30,32,33]. These ion channels are believed to play an important role in asthma as well as in COPD [34,35,36]. Asthma is an inflammatory disease and many hopes have been attached to TRP antagonists as potential asthma relievers, though the research in this field is not unison [37]. However, a recent study, in a mice model of allergic asthma, also showed that a TRPV1 inhibitor decreased airway inflammation, immunoglobulin E (IgE) levels and airway hyperreactivity [38].

In addition, non-neuronal TRPV1 channels may be involved in airway disorders, and epithelial cells play a significant role in both asthma and COPD. McGarvey et al. recently found increased epithelial TRPV1 expression in severe asthma, indicating that the TRPV1 channels could represent a possibility to treat severe asthma where available medications have not been successful [29].

## 3. TRP Ion Channels in Chronic Cough

There is increasing evidence of the role of TRP ion channels, expressed by C and Aδ fibers in the cough mechanism. The cough reflex is induced by activation of airway sensory nerves and TRP ion channels related to the vanilloid (TRPV) and the ankyrin (TRPA) families [33,39,40,41]. In thermal nociception and in inflammatory hyperalgesia, the TRPV1 is an integrator of triggering stimuli and plays a role in protective reflexes like coughing and sneezing. In CIC, increased expression of TRPV1 was found and also a correlation between cough sensitivity to inhaled capsaicin and the quantity of TRPV1-positive nerves [42,43]. Several studies have pointed to heightened capsaicin cough sensitivity in CIC [14,44]. Capsaicin is the main, often used agonist for TRPV1; as an inhalant, it has for decades been used in cough provocation, regarded as a safe and reproducible procedure [2,11,45,46,47,48,49,50]. The results from such cough provocation studies suggest that the pathophysiology of CIC is related to airway mucosal TRP receptors in sensory nerves, reacting to noxious stimuli [33], and today there is a common opinion that the “cough without explanation” could be regarded as a neuropathic disorder [21,23]. Whether the main mechanisms in CIC are generally peripherally or centrally controlled is, however, debated [51,52,53], though both peripheral and central mechanisms may be involved.

### 3.1. TRP Ion Channels as Therapeutic Targets for CIC

In recent years, there has been an emerging interest in the family of TRP ion channels as possible therapeutic targets for a number of airway diseases, among them CIC [31,37,54]. The focus has been not only on TRPV1 but also on TRPA1 and TRPM8 [55]. Modulation of these TRP ion channels may be followed by disease improvement in a variety of airway disorders including CIC [34,54].

#### 3.1.1. TRPV1 as a Therapeutic Target for CIC

TRPV1 is, in addition to being involved in cough and rhinitis, a major actor in pain and pain sensitivity, subsequently followed by increasing interest in the development of TRPV1 antagonists, both for cough treatment and for neuropathic pain disorders [37,56,57]. For the treatment of pain, there have long been several products available (creams and patches) targeting the TRPV1, using topical capsaicin to desensitize the sensory C fibers, probably by “exhausting” signal substances of the sensory nerves [57]. A recent study showed that higher concentration of capsaicin in patches provided better relief of neuropathic and chronic pain [58]. Topical treatment with capsaicin solution may also reduce symptoms in non-allergic chronic rhinitis [59]. A current study found, in such patients, increased levels of substance P in nasal lavage and overexpression of TRPV1 in nasal mucosa and treatment with topical capsaicin decreased symptoms and lowered nasal hyperreactivity [60]. The authors hypothesized that, in the nasal mucosa, capsaicin ablated the TRPV1–substance P nociceptive signaling pathway.

The TRPV1 ion channel was initially also called the “capsaicin receptor”, due to capsaicin’s close relation to this receptor [61]. The noxious effect of capsaicin in chili fruits is well known and is used in spices and pepper spray [62].

In light of the current lack of effective cough medications, it is natural that a number of commercial pharmaceutical companies are developing drugs acting as antagonists on TRP ion channels [37]. Resolvin D2 is a potent endogenous antagonists for TRPV1 [63], and there have been many exogenous TRPV1 antagonists identified, some of them synthetic analogs of capsaicin, such as capsazepine [55]. There have been hopeful findings in animal testing [64], but some of these projects seem to have problems when the medication is finally tested in humans beings, having adverse effects including hyperthermia and impaired noxious heat sensation, which has been extensively reviewed earlier [31,37,65,66]. A recent study on the TRPV1 antagonist SB-705498 did reduce the capsaicin cough sensitivity in patients with chronic cough, but not the cough frequency [65]. Up to now, there has been no oral TRPV1 antagonist available on the market for either cough or pain.

Desensitization is a complex, not exactly defined process, but it has a therapeutic potential and when inhaled, capsaicin in humans is known to cause a short period of desensitization in terms of less cough sensitivity [67,68].

Capsaicin, the major trigger of TRPV1, is found naturally in a great variety of food dishes comprising different kinds of chili products, giving a “hot” taste and further inducing a number of physiological reactions of which some seem to be health promoting [69]. The use of chili in food varies between different countries and cultures. Most western countries have no long tradition of the use of chili in cooking. A dish with a lot of hot chili can result in undesired symptoms like irritation in the mouth and throat, sneezing, eye irritation, and sometimes coughing. It is “common knowledge” that it is possible to get used to spicy food by gradually increasing the intake. The TRPV1 receptors use neuropeptides to evoke brain signals, and if these receptors are regularly stimulated, neuropeptides are depleted, and few or no symptoms are awakened by spicy food [37,70]. Previously, it was thought that capsaicin desensitization is only possible when capsaicin is applied locally on skin or inhaled. For ingested capsaicin to have an effect on coughing, it must act systemically after transport in the circulatory system. Little is known about the absorption and distribution of capsaicin in humans, and only one study has looked at capsaicin human pharmacokinetics—after a large meal of Thai capsicums [71]. This study found a low bioavailability of capsaicin, though this is likely explained by conversion of capsaicin in the intestine to dihydrocapsaicin, an intestinal metabolite of capsaicin, which was not measured but probably induced reactions similar to those from capsaicin. Given the interest in capsaicin, both for the purpose of cough and pain suppression and also as an emerging therapy for obesity and cancer [31,69,72], this is a major knowledge gap. A method developed to analyze capsaicin in human sera with high performance liquid cromatography (HPLC) gives new possibilities of reducing this gap and studying any dose-response relation [73].

In the clinic, we have encountered patients who claim to have “treated” their cough by eating very spicy food equivalent to several fresh chili pepper fruits per day. However, the same dietary recommendations have not been feasible because of the experience of the strong flavor. In a recent pilot study, 21 patients with chronic cough had fewer symptoms and reduced cough reflex sensitivity if they regularly consumed capsules containing concentrated capsaicin from chili peppers [74]. There were no adverse effects and the daily intake of capsaicin corresponded to what it is common to eat regularly in countries such as Mexico and Thailand. Epidemiological research found the incidence of chronic cough in countries with regular intake of spicy food to be around 2%, compared to up to 20% in western countries [75], supporting the observation that first led to this work in Sweden. Since the current pilot study [74] has showed convincing results, orally given capsaicin has been identified as a possible treatment of cough, offering a good option for those people not used to spicy foods.

#### 3.1.2. TRPM8 as a Therapeutic Target for CIC

Patients with CIC and SHR often complain that exercising in cold air is an inducing factor for cough [47,48,76,77], and exercise in a cold air chamber was followed both by both coughing and increased capsaicin sensitivity [78]. It seems likely that TRPM8 and TRPA1, known to react to low temperatures, are involved in airway symptoms induced by cold air. The sensation of cold evoked by menthol was explained by the discovery of the TRPM8 ion channel reacting to cool temperatures and menthol [79,80,81]. Menthol (C_10_H_20_O), synthetically produced or extracted from mint oils, is a covalent organic compound that is present in a number of over the counter (OTC) products for ameliorating symptoms in rhinitis, common cold and throat irritation. Eccles et al. found no significant effect on nasal patency [82]. Whereas OTC products comprising menthol for relief of airway symptoms have been available for decades, only a few scientific studies support the cough-relieving effects from menthol products, though the interest in a potential effect in cough treatment seems to be increasing.

Menthol is also used in the tobacco industry as a cigarette brand to improve flavor and disguise the airway irritation evoked by smoking [83,84]. Already in 1994, Morice et al. published results from a study proving that, in humans, cough induced by inhaling citric acid could be prevented from *pre* inhalation of menthol [85]. A year later, the concordant results were shown in guinea pigs [85]. However, in children, Kenia et al. found no difference in cough count compared to placebo when a provocation with citric acid was preceded by inhalation of menthol, whereas the perception of nasal patency increased [86]. Another study showed that premedication of menthol inhalation before bronchoscopy did not improve coughing during the process, but late symptoms of cough and dyspnea improved as did peak expiratory flow [87]. Later reports indicated the possibility of reducing cough sensitivity with inhaled or intranasal menthol given before a provocation with cough-inducing agents [88,89,90,91]. In summary, menthol seems to have a capacity to reduce the sensitivity of an important airway defense mechanism that could be used for good (in cough medications) or for bad (in cigarette brands) [84].

Also regarding TRPM8 and menthol, there is a parallel between the airways and the skin regarding the treatment of itch and pain, with some studies reporting a beneficial effect from topical menthol preparations [92,93,94]. However, in healthy humans, topical cutaneous menthol provoked cold allodynia, suggested as being the results from a sensitization of nociceptors reacting on cold stimuli [95,96], indicating complex innervation mechanisms where menthol in some situations may be hyperalgesic but may be analgesic in some patients with peripheral and central neuropathic pain. Also illustrating the confusing role of menthol and the TRP channels are the findings that the TRPA1 ion channel, known to evoke cough from noxious stimuli and cold, is a highly sensitive receptor for menthol, probably involved in a variety of menthol induced physiological reactions [36,97]. Takaishi et al. elucidated these questions, demonstrating a reciprocal effect of capsaicin and menthol wherein menthol proved to have an anti-nociceptive effect on TRPV1, and capsaicin inhibited TRPM8-mediated currents [98]. Furthermore, there was a mutual inhibition of temperature activation in human TRPV1 or TRPM8 and a binding site of menthol was identified in TRPV1.

Although it is better understood today, the theoretical explanation as to why menthol has an ameliorating effect on cough reflex sensitivity remains in part obscure, but acting via TRPM8, menthol may interfere with TRPV1 and the cough outcome from capsaicin and environmental irritants [34,35].

## 4. Conclusions

During the last decade, a new paradigm has been developed of CIC as a possible neuropathic disease that could be linked to the TRP ion channels, with persisting cough as an unmistakable symptom. The lack of effective medical treatment in CIC is obvious and frustrating, though neuromodulators and new receptor antagonists indicate different novel options to ameliorate cough and cough sensitivity, as does the possibility of TRPV1 desensitization [23,74,99]. The TRPV1 antagonist SB-705498 revealed no negative properties but a somewhat surprising effect only on the capsaicin cough sensitivity, not on the cough symptoms [65]. However, this is in concordance with other reports studying rhinitis and pruritus [100,101,102,103] showing no improvement from treatment with SB-705498. The results could suggest that TRPV1 may not be of such great importance in chronic cough as earlier believed, but the evident relation between chronic cough, TRPV1 expression and cough sensitivity to inhaled capsaicin contradicts such a paradigm change. The SB-705498 is a highly selective molecule and the blocking of TRPV1 in terms of both lowering capsaicin sensitivity and improving cough symptoms may demand a more complex structure interacting on different binding sites. It would, however, be interesting to carry out a clinical study with SB-705498 in patients with severe, refractory asthma, since the mechanisms in such asthma is quite different from those in chronic cough and recent findings showed increased epithelial TRPV1 expression in this difficult to treat condition [29]. One major problem is the lack of tools to study how TRP channels appear and change in CIC and other airway disorders.

There is a rich “flora” of OTC medications based on a diversity of substances, though few scientific studies can confirm measurable effects. Future research in cough medication should focus on proving reliable effects with few adverse events.
